# Influence of the ratio of gate length to drain-to-source distance on the electron mobility in AlGaN/AlN/GaN heterostructure field-effect transistors

**DOI:** 10.1186/1556-276X-7-434

**Published:** 2012-08-03

**Authors:** Yuanjie Lv, Zhaojun Lin, Lingguo Meng, Chongbiao Luan, Zhifang Cao, Yingxia Yu, Zhihong Feng, Zhanguo Wang

**Affiliations:** 1 School of Physics, Shandong University, Jinan, 250100, China; 2 Science and Technology on ASIC Laboratory, Hebei Semiconductor Research Institute, Shijiazhuang, 050051, China; 3 Laboratory of Semiconductor Materials Science, Institute of Semiconductors, Chinese Academy of Sciences, Beijing, 100083, China

**Keywords:** electron mobility, drain-to-source distance, AlGaN/GaN heterostructures, polarization Coulomb field scattering

## Abstract

Using measured capacitance-voltage curves with different gate lengths and current–voltage characteristics at low drain-to-source voltage for the AlGaN/AlN/GaN heterostructure field-effect transistors (HFETs) of different drain-to-source distances, we found that the dominant scattering mechanism in AlGaN/AlN/GaN HFETs is determined by the ratio of gate length to drain-to-source distance. For devices with small ratio (here, less than 1/2), polarization Coulomb field scattering dominates electron mobility. However, for devices with large ratio (here, more than 1/2), longitudinal optical (LO) phonon scattering and interface roughness scattering are dominant. The reason is closely related to polarization Coulomb field scattering.

## Background

Owing to potential applications in high power and high frequency electronic devices associated with outstanding material properties, AlGaN/GaN heterostructure field effect transistors (HFETs) have attracted extensive research to improve the device performance [1-3]. The strained AlGaN/AlN/GaN heterostructure with a thin AlN interlayer has been the popular material structure for AlGaN/GaN HFETs due to the improved transport properties of two-dimensional electron gas (2DEG) and electron mobility [4,5]. According to our former report, it is found that the ratio of gate length to drain-to-source distance has an important influence on electron mobility and determines the dominant scattering mechanism in the AlGaN/AlN/GaN HFETs with the drain-to-source distance of 100 μm [6]. However, the above influence on the electron mobility in AlGaN/AlN/GaN HFETs with different drain-to-source distances has not been investigated. Meanwhile, mainstream microwave power AlGaN/AlN/GaN HFETs are in small-size drain-to-source distances [7]. Therefore, it is of great importance to investigate the influence of the ratio of gate length to drain-to-source distance on the electron mobility in AlGaN/AlN/GaN HFETs with different drain-to-source distances. In this study, rectangular AlGaN/AlN/GaN HFETs with different drain-to-source distances and gate geometrical areas were fabricated, and the influence of the ratio of gate length to drain-to-source distance on the electron mobility in AlGaN/AlN/GaN HFETs with different drain-to-source distances was investigated.

## Methods

The heterostructure layer employed in this study was grown by molecular beam epitaxy on a (0001) sapphire substrate. The structure consists of a 40-nm AlN nucleation layer, followed by a 3-μm undoped GaN layer, a 0.5-nm AlN interlayer, and a 22.5-nm-thick undoped Al_0.28_ Ga_0.72_ N layer. Hall measurements indicated a sheet carrier density of around 1.1 × 10^13^ cm^−2^ and an electron mobility of 1,800 cm^2^/V·s at room temperature. For device processing, mesa isolation was performed using Cl_2_/BCl_3_ reactive ion etching. The source and drain ohmic contacts were formed by depositing Ti/Al/Ni/Au using e-beam evaporation and lift-off and then were annealed in a rapid thermal annealing system. With transmission line method patterns, the specific resistivity of the contacts was measured to be 7 × 10^−5^ Ω·cm^2^. The source and drain contacts were rectangular: 100 μm wide and 50 μm long. Drain-to-source distances with 60, 20, 15, and 9 μm were prepared. Ni/Au (60/160 nm) Schottky contacts of varying areas were then deposited symmetrically in the middle between the source and drain ohmic contacts by e-beam evaporation. The Schottky contact sizes in AlGaN/AlN/GaN HFETs with a 60-μm drain-to-source distance are 12/100 (length/width), 24/100, 36/100, and 48/100 μm which are marked as 60-a, 60-b, 60-c, 60-d, respectively. The Schottky contact sizes in AlGaN/AlN/GaN HFETs with a 20-μm drain-to-source distance are 4/100 (length/width), 8/100, 12/100, and 16/100 μm which are marked as 20-a, 20-b, 20-c, 20-d, respectively. Schottky contacts of 3/100 μm (length/width) were deposited in AlGaN/AlN/GaN HFETs with 15- and 9-μm drain-to-source distances which are marked as 15-a and 9-a, respectively. Capacitance-voltage (*C*-*V*) measurements were performed at room temperature using an Agilent B1520A (Agilent Technologies, Inc., Santa Clara, CA, USA) at 1 MHz, and current–voltage (*I*-*V*) measurements for the AlGaN/AlN/GaN HFETs were also performed at room temperature using an Agilent B1500A semiconductor parameter analyzer.

## Results and discussion

**Figure 1:**
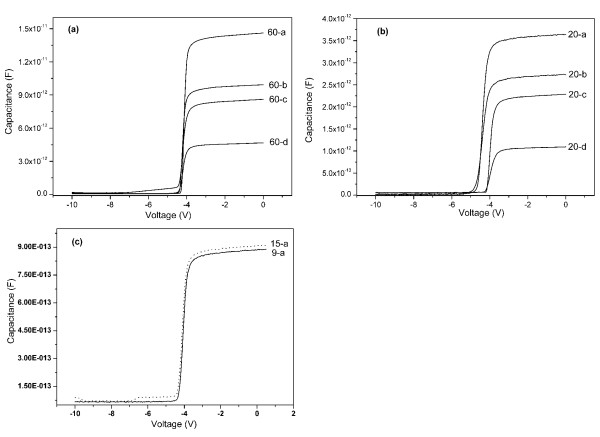
**Measured*C*-*V*curves at room temperature of the Ni Schottky contacts.** With different areas for the devices with drain-to-source distances of 60 (**a**), 20 (**b**), and 15 and 9 μm (**c**).

**Figure 2:**
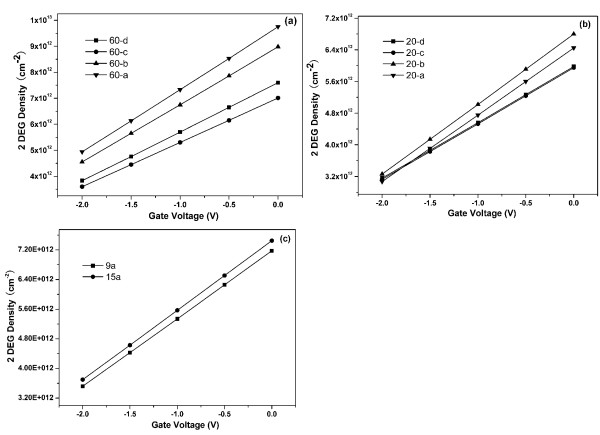
**Calculated 2DEG electron density*n***_**2D**_**under different gate biases.** They are for samples with drain-to-source distances of 60 (**a**), 20 (**b**), and 15 and 9 μm (**c**).

Figure 1 shows the *CV* curves of the Ni Schottky contacts with different areas for the devices with drain-to-source distances of 60, 20, 15, and 9 μm, respectively. The *CV* measurements were obtained using the source contact and the Ni Schottky contact. The different threshold voltages of samples 20-c and 20-d with respect to samples 20-a and 20-b were due to the different strains under the Schottky contacts caused by the different Schottky contact areas [8]. The 2DEG electron density (*n*_2D_) under different gate biases with different Ni Schottky contact areas can be obtained by the integration of the measured *CV* curves [9], and the calculated results are shown in Figure 2.

**Figure 3:**
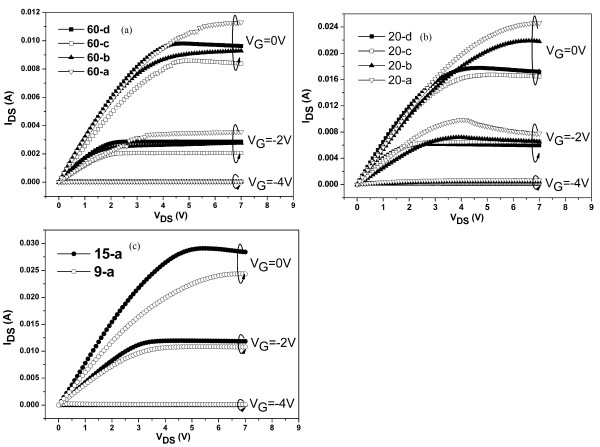
**Measured*I*-*V*curves at room temperature for samples with different drain-to-source distances.** 60 (**a**), 20 (**b**), and 15 and 9 μm (**c**).

**Figure 4:**
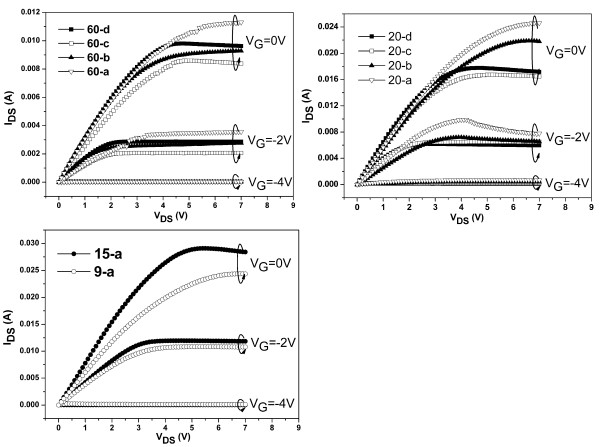
**Relationship between electron mobility of 2DEG and applied gate bias at room temperature.** They are for samples with drain-to-source distances of 100 (**a**), 60 (**b**), 20 (**c**), and 15 and 9 μm (**d**); (**a**) is referenced from our former literature [6].

The *IV* characteristics for the rectangular AlGaN/AlN/GaN HFETs with different drain-to-source distances were measured and shown in Figure 3. The saturation current is higher with shorter gate length due to the long distance between the drain and gate edge. The abnormality of 60-d and 20-d may be due to the nonuniformity of the AlGaN/GaN heterostructures or the ohmic contacts. The electron mobility of the 2DEG in the strained AlGaN/AlN/GaN heterostructures can be calculated with the *IV* characteristics and 2DEG electron density as described in [6]. Due to the low specific resistivity of the ohmic contacts (7 × 10^−5^ Ω·cm^2^), the source and drain resistances were ignored during the calculation. The calculated results are shown in Figure 4. Also, the 2DEG electron mobility of the rectangular AlGaN/AlN/GaN HFETs with drain-to-source of 100 μm in [6] was also shown in Figure 4a, and from the smallest to the largest in Schottky contact sizes, they are marked as 100-a, 100-b, 100-c, and 100-d.

As one can see from Figure 4, the 2DEG electron mobility increases with gate voltage for the devices with small ratio of gate length to drain-to-source distance (here, less than 1/2),but it decreases for the one with large ratio (here, more than 1/2). It is well known that there are mainly five kinds of important scattering mechanisms to affect the 2DEG electron drift mobility in AlGaN/GaN HFET samples, and these scattering mechanisms are ionized impurity scattering [9], dislocation scattering [10], polarization Coulomb field scattering [6,9], longitudinal optical (LO) phonon scattering, and interface roughness scattering [11]. The ionized impurity scattering and dislocation scattering can be ignored in our samples as discussed in [6]. The variety of electron mobility according to the gate bias can be explained as follows.

The Schottky gate produced a partial strain relaxation in the AlGaN layer, and then the polarization charges at AlGaN/AlN interface are distributed irregularly (spatial correlation is only partial) [8]. Thus, an additional scattering potential (polarization Coulomb field scattering potential) in comparison with the un-gated heterostructure is formed. For polarization Coulomb field scattering, the electron mobility rises with the increasing electron density, but it decreases for the LO phonon scattering and the interface roughness scattering [6,9]. For the devices with large ratio of gate length to drain-to-source distance, the gradient of the polarization charge density is relatively small; therefore, the scattering associated with polarization Coulomb field is relatively weak [6]. As a result, the LO phonon scattering and the interface roughness scattering dominate the 2DEG electron mobility, leading to the monotonic decrease for the mobility. For the devices with small ratio of gate length to drain-to-source distance, the gradient of the polarization charge density is large. Thus, the polarization Coulomb field scattering is the dominant carrier scattering mechanism, which results in the monotonic increase for the mobility of the 2DEG electrons with gate voltage. For a given gate bias, the electron mobility of the 2DEG decreases with the reducing Ni Schottky contact area as shown in Figure 4; this can be explained by the weaker polarization Coulomb field scattering corresponding to the larger Ni Schottky contact area. Therefore, the conclusion can be made that the dominant scattering mechanism in the AlGaN/AlN/GaN HFETs is determined by the ratio of gate length to drain-to-source distance. With the ratio of less than 1/2, the polarization Coulomb field scattering dominates the 2DEG electron mobility in the AlGaN/AlN/GaN HFETs, while with the ratio larger than 1/2, the LO phonon scattering and the interface roughness scattering are dominant in the devices.

## Conclusions

In summary, Ni Schottky contacts of different geometrical areas were deposited on strained AlGaN/AlN/GaN heterostructures with different drain-to-source distances. With the measured *C*-*V* curves and the *I*-*V* characteristics of AlGaN/AlN/GaN HFETs, we have investigated the influence of the ratio of gate length to drain-to-source distances on the electron mobility of the 2DEG in rectangular AlGaN/AlN/GaN HFET devices. We found that the dominant scattering mechanism in the AlGaN/AlN/GaN HFETs is determined by the ratio of gate length to drain-to-source distance. For the devices with small ratio (here, less than 1/2), the polarization Coulomb field scattering dominates the 2DEG electron mobility. For the devices with large ratio (here, more than 1/2), the LO phonon scattering and the interface roughness scattering are dominant.

## Competing interests

The authors declare that they have no competing interests.
